# Exogenous application of sulfur-rich thiourea (STU) to alleviate the adverse effects of cobalt stress in wheat

**DOI:** 10.1186/s12870-024-04795-1

**Published:** 2024-02-21

**Authors:** Aiman Zahid, Kaleem ul din, Muhamad Ahmad, Umer Hayat, Usman Zulfiqar, Syed Muhammad Hassan Askri, Muhammad Zohaib Anjum, Muhammad Faisal Maqsood, Nazish Aijaz, Talha Chaudhary, Hayssam M. Ali

**Affiliations:** 1 Department of Botany, University of Agriculture, Faisalabad, 38040 Pakistan; 2 Department of Agronomy, University of Agriculture, Faisalabad, 38040 Pakistan; 3 Department of Agronomy, Faculty of Agriculture and Environment, The Islamia University of Bahawalpur, Bahawalpur, 63100 Pakistan; 4 Zhejiang Key Laboratory of Crop Germplasm Resource, Department of Agronomy, College of Agriculture and Biotechnology, Zhejiang University, Hangzhou, 310058 China; 5 Department of Forestry and Range Management, University of Agriculture, Faisalabad, 38040 Pakistan; 6 Department of Botany, The Islamia University of Bahawalpur, Bahawalpur, 63100 Pakistan; 7 School of Biomedical Science, Hunan University, Changsha, Hunan China; 8 MOA Key Laboratory of Soil Microbiology, Rhizobium Research Center, China Agricultural University, Beijing, China; 9 Faculty of Agricultural and Environmental Sciences, Hungarian University of Agriculture and Life Sciences 2100, Godollo, Hungary; 10 Department of Botany and Microbiology, College of Science, King Saud University, Riyadh, 11451 Saudi Arabia

**Keywords:** Antioxidants, Cobalt stress, Secondary metabolites, Thiourea, Wheat

## Abstract

Heavy metal stress affects crop growth and yields as wheat (*Triticum aestivum* L.) growth and development are negatively affected under heavy metal stress. The study examined the effect of cobalt chloride (CoCl_2_) stress on wheat growth and development. To alleviate this problem, a pot experiment was done to analyze the role of sulfur-rich thiourea (STU) in accelerating the defense system of wheat plants against cobalt toxicity. The experimental treatments were, i) Heavy metal stress (a) control and (b) Cobalt stress (300 µM), ii) STU foliar applications; (a) control and (b) 500 µM single dose was applied after seven days of stress, and iii) Wheat varieties (a) FSD-2008 and (b) Zincol-2016. The results revealed that cobalt stress decreased chlorophyll *a* by 10%, chlorophyll *b* by 16%, and carotenoids by 5% while foliar application of STU increased these photosynthetic pigments by 16%, 15%, and 15% respectively under stress conditions as in contrast to control. In addition, cobalt stress enhances hydrogen peroxide production by 11% and malondialdehyde (MDA) by 10%. In comparison, STU applications at 500 µM reduced the production of these reactive oxygen species by 5% and by 20% by up-regulating the activities of antioxidants. Results have revealed that the activities of SOD improved by 29%, POD by 25%, and CAT by 28% under Cobalt stress. Furthermore, the foliar application of STU significantly increased the accumulation of osmoprotectants as TSS was increased by 23% and proline was increased by 24% under cobalt stress. Among wheat varieties, FSD-2008 showed better adaptation under Cobalt stress by showing enhanced photosynthetic pigments and antioxidant activities compared to Zincol-2016. In conclusion, the foliar-applied STU can alleviate the negative impacts of Cobalt stress by improving plant physiological attributes and upregulating the antioxidant defense system in wheat.

**Graphical Abstract:**

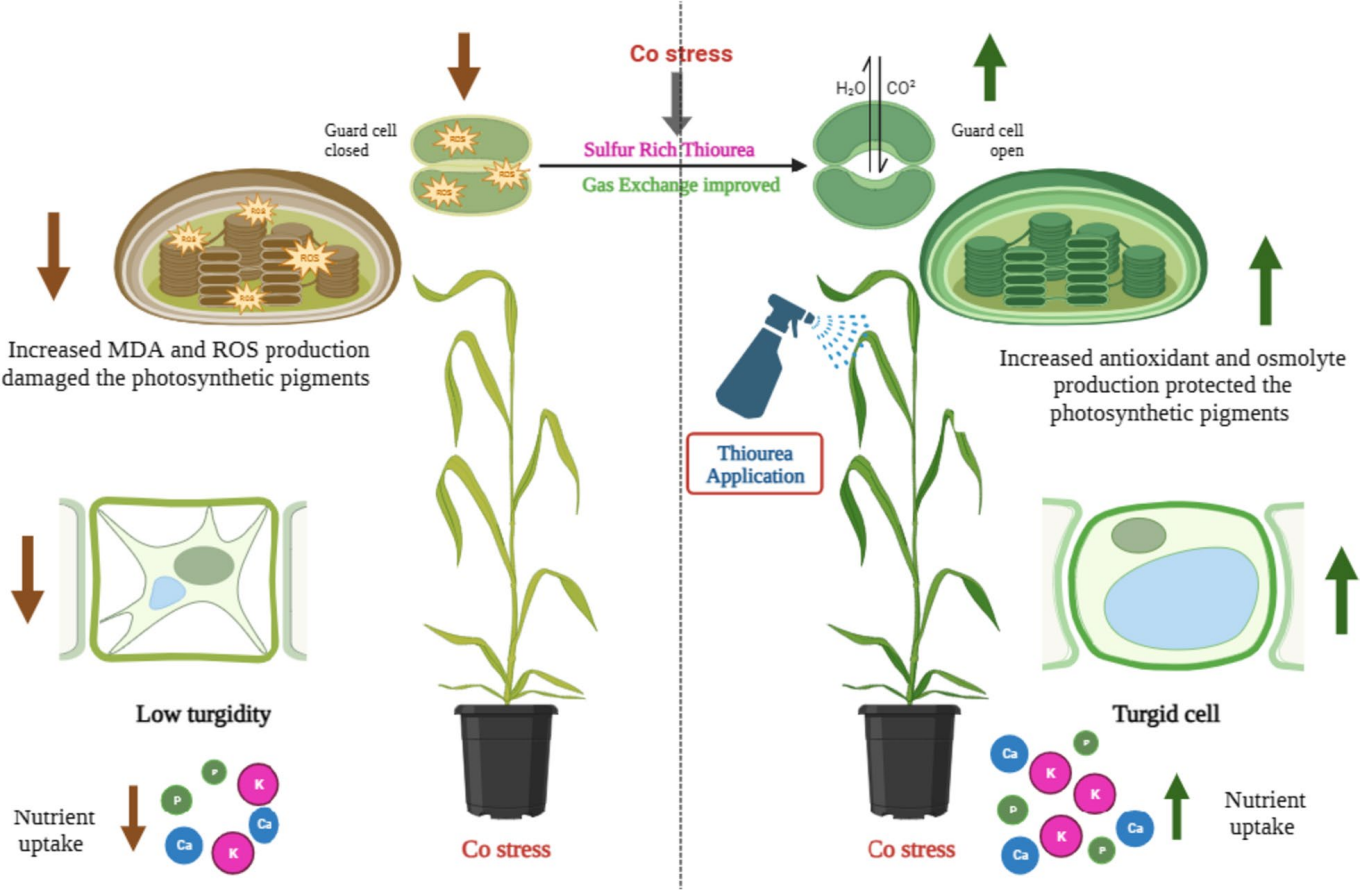

## Introduction

 Wheat (*Triticum aestivum* L.) is a mostly cultivated grain crop all over the world that contains essential proteins, carbohydrates, and major dietary fibers that are essential for a healthy life [1]. Crop productivity is significantly affected by drought, salinity, cold, heat and heavy metals stress [2–5]. Wheat crops are sensitive to heavy metal stresses [6] which alter most of the biochemical responses with reactive oxidative species (ROS) overproduction and lead to disturbance in the electron transport chain that causes restricted growth and yield loss [7, 8].

Cobalt (Co) is one of the heavy metals that, in excess concentration, may seriously harm plant cells, reducing biomass and growth by altering the structure of the root [9]. Various anthropogenic activities, industrial production, and urbanization processes are linked to the release of cobalt in the soil. These include surface runoff, volcanic eruptions, burning fossil fuels, smelting and refining copper and nickel, making alloys, producing batteries, and using phosphate fertilizers in agriculture [10]. The majority of industrial and transportation areas are the ones most susceptible to the occurrence of high cobalt contents in soil of anthropogenic origin. High cobalt-content soils are frequently found close to metal smelting, machinery manufacturing, and mining operations [11]. Like other heavy metals, Co causes cell damage and decreases plant growth and yield by upregulating the Haber-Weiss and Fenton processes, which result in the production of reactive oxygen species. Higher concentrations of Co destabilize multiple metabolic pathways and induce oxidative damage to biomolecules, resulting in lipid peroxidation, membrane degradation, and protein carboxylation [12]. Cobalt-enhanced levels in plants distort chloroplast structure, ultimately leading to disruption in carbon dioxide assimilation due reduction in the uptake of carbon [13]. Enzymes used in the biosynthetic pathway of chlorophyll were disturbed by the distortion in the structure of rubisco (ribulose-1,5-bisphosphate-carboxylase/oxygenase) due to the replacement of Mg atom by Co in rubisco that is a crucial protein for the photosynthetic process [14]. Reactive oxygen species (ROS) like superoxide anion radicals (O^2−^) and hydrogen peroxide (H_2_O_2_) are produced when cobalt toxicity interacts with molecules of oxygen and electrons that escape from the photosynthetic electron transfer system [15]. Plants possess various defense mechanisms that play a vital role in providing tolerance against various abiotic stresses (Co) by altering various physiological functions. These activities include the build-up of sugars, which actively regulates growth, protein synthesis, carbon partitioning, amino acid and lipid metabolism, and osmotic homeostasis [16]. Plants have developed a defense mechanism to counteract oxidative stress caused by heavy metals. However, low antioxidant concentrations can cause plant cells to be unable to squelch dangerous reactive oxygen species (ROS), which can lead to reduced growth and yield [17].

Applying mineral nutrients or bio-regulators, which control multiple physiological and biochemical mechanisms at the metabolic and whole plant levels, improves plants’ natural defense against abiotic stress [18]. Thiourea is a sulfur-rich plant growth promoter that modulates plant development and effectively prevents the plants from oxidative damage imposed by abiotic stress [19, 20]. It is a non-physiological thiol-based ROS scavenger that contains sulfur (S) 42% and nitrogen (N) 36% [21] and can lower the stress-prompted redox imbalance and different injuries of the plant [22, 23]. Exogenously applied STU enhances the stress tolerance of crops [24, 25]. Causing an increase in growth and crop productivity, membrane stability, antioxidant potential, and photosynthetic efficiency [26, 27]. Several studies have reported that STU application plays a significant role in coping with a variety of abiotic stress by improving the morpho-physiological, biochemical, and yield contribution indices in several crops such as wheat [28–30], maize [31], canola [32], camelina [33, 34], and barley [35].

The exogenous application of STU to lower the negative effects of abiotic stress has been reported in previous studies. However, the role of STU in alleviating the toxic effects of Co stress in the different wheat varieties is limited and requires further investigation. Therefore, this study hypothesized that STU applications may alleviate the toxic effects of Co stress in wheat. The current investigation was conducted to evaluate the ameliorative role of STU to plant defense systems under Co stress by improving plant physiological attributes and antioxidant activities in wheat.

## Materials and methods

The seeds of wheat varieties, FSD-2008 and Zincol-2016, were procured from the Ayub Agricultural Research Institute (AARI) in Faisalabad, Pakistan. A pot study planned to explore the protective role of thiourea on wheat plants grown under Cobalt chloride stress in the Old Botanical Garden wire house, Department of Botany, University of Agriculture Faisalabad (31° 25‘N, 73° 05’E), Pakistan. The experimental study was performed under a Completely Randomized Design (CRD) with the three-factor factorial design having three replicates. The experimental treatments were, i) Heavy metal stress; (a) control and (b) Cobalt stress (300 µM) [15] applied to sand-filled pots through irrigational water, ii) STU foliar applications; (a) control, and (b) STU (500 µM) was applied after seven days of stress, and iii) Wheat varieties (a) FSD-2008 and (b) Zincol-2016. The diameter and height of each plastic pot used in the experiment were 31 cm and 25 cm, respectively. The 8 kg purely washed sand was used to fill each plastic pot. Wheat seeds (5 g) of both varieties were disinfected with sodium hypochlorite solution by using a method proposed by Smilanick et al. [36] then ten seeds of each wheat variety were sown in each pot. The duration of the experiment was 60 days. The half-strength Hoagland nutrient solution prepared by using the method of Arnon and Hoagland [37] is applied on germination and at every 10-day interval to fulfill the nutrient needs of the crop. Thinning was done on the second leaf stage and after thinning only eight wheat seedlings in each pot were maintained for further study. The cobalt chloride (300 µM) solution was prepared in 12 L of half-strength Hoagland solution. One liter (300 µM CoCl_2_) solution applied was 30 days after sowing (DAS) (BBCH growth stage code-30; Principal growth stage-3: Stem elongation) [38, 39] to each pot wheat seedlings to develop stress conditions. To the control plants, only half half-strength Hoagland solution was applied to maintain growth conditions. The one-liter STU (500 µM) solution was prepared and filled in a plastic bottle. After seven days of stress conditions, the STU (500 µM) single dose was applied as a foliar spray at 38 DAS (BBCH growth stage code-38; Principal growth stage 3: Flag leaf just visible, still rolled) [38, 39] on wheat plant seedlings. The 10 mL foliar spray was applied on each plastic pot wheat seedlings with the help of a plastic bottle sprayer in a way that all seedlings were wet with STU solution. Three replicates are taken for the measurement of each attribute.

## Determination of morphological attributes

Plants were harvested after 60 DAS to determine growth attributes i.e. shoot and root length, fresh and dry weights, and leaf area. Seedling and root length were determined with the help of a calibrated meter rod. After the measurement of fresh weight, the shoots and roots were sun-dried for 96 h and then kept in an electric oven at 72 °C until constant weight and the dry weight were measured with an electronic balance.

## Determination of photosynthetic pigments

Chlorophyll and carotenoid contents of wheat leaves were determined at 60 DAS according to the method of Arnon [40].

### Determination of hydrogen peroxide and malondialdehyde

The sampling was done at 60 DAS to determine H_2_O_2_ and MDA. The activity of H_2_O_2_ was measured by Velikova et al. [41] described a method to determine the MDA protocol used by Heath and Packer, [42] was followed.

### Determination of enzymatic antioxidant activities

The samples to determine antioxidant activities were taken at 60 DAS. According to the method mentioned by Chance and Maehly [43] with few modifications peroxidase (POD) and catalase (CAT) activity were measured. Giannopolitis and Ries [44] described a protocol that was followed to determine superoxide dismutase (SOD) enzyme activity.

### Determination of non-enzymatic antioxidants

The samples to determine non-enzymatic antioxidant activities were taken at 60 DAS. Kim et al. [45] proposed a method used for flavonoid determination. Mukherjee and Choudhuri [46] described a method for the determination of ascorbic acid was followed. The anthocyanin determination method was given by Stark et al. [47]. The determination of phenolic compounds was done by following the protocol given by Noreen et al. [48].

### Determination of osmo-protectants

The samples to determine osmo-protectants activities were taken at 60 DAS. Handle [49] method used for total soluble sugar determination. Total soluble protein analysis was performed by following the Bradford [50] method. Proline in plants was measured by following the method of Bates et al. [51].

### Determination of mineral nutrients

The samples to determine the uptake of mineral nutrients in shoots of wheat plants were taken at 60 DAS. Shoot and root ionic contents were measured by protocol given by Allen et al., [52]. Phosphorous content was measured by using the same extract according to the method of Jackson, [53] with the help of a spectrophotometer.

### Plant guidelines

All the plant experiments were performed by relevant institutional, national, and international guidelines and legislation.

### Statistical analysis

The experiment involved three replications using a complete randomized design (CRD), with data analysis using Statistix software (8.1version) and Microsoft Excel-2016 for figures. Pearson’s correlation, clustered heatmap, and PCA were performed among different traits using software like Origin pro-2022 and R-Studio.

## Results

### Morphological attributes

Morphological attributes were significantly affected under the cobalt stress and STU foliar application in wheat varieties. The interaction among cobalt stress × STU applications × wheat varieties was non-significant for morphological attributes. Results have revealed that cobalt stress decreased the shoot length (40%), root length (52%), shoot fresh weight (32%), root fresh weight (37%), shoot dry weight (24%), root dry weight (50%), and leaf area index by (50%) in comparison to control (Table 1). The wheat variety Zincol-2016 showed more reduction in growth parameters as compared to FSD-2008. However, STU foliar applications played an ameliorative role in reducing the detrimental effect of cobalt stress in wheat varieties and improved the morphological attributes. The shoot fresh weight was improved (15%) and root fresh weight was improved by 14% in wheat plants grown under cobalt stress. Among the cultivates, FSD-2008 showed better performance in comparison to Zincol-2016 under cobalt stress and STU applications in terms of morphological parameters as shoot length and root length increased (14%) and (15%) respectively in FSD-2008 as compared to Zincol-2016.

**Table 1:** Effect of foliar applied STU on the morphological indices of the wheat cultivars under cobalt stress conditions

Cultivars	Treatments	Shoot fresh weight (mg/g FW)	Root fresh weight (mg/g FW)	Shoot dry weight (mg/g FW)	Root dry weight (mg/g FW)	Shoot length (cm)	Root length (cm)	Leaf area (cm 2)
**FSD-2008**	Control	4.82 ± 0.2ab	0.40 ± 0.01abc	0.62 ± 0.02ab	0.17 ± 0.01ab	61.1 ± 2.1ab	16.3 ± 0.1b	30.4 ± 0.1abcd
	STU	5.28 ± 0.2ab	0.45 ± 0.01ab	0.7 ± 0.02a	0.20 ± 0.01ab	65.6 ± 1.1ab	20.6 ± 1.1ab	34.2 ± 0.1ab
	Co	3.64 ± 0.2 cd	0.29 ± 0.02de	0.45 ± 0.03bc	0.08 ± 0.01c	45.3 ± 1.1 cd	10 ± 0.4c	22.8 ± 1.0d
	STU + Co	5.65 ± 0.2a	0.48 ± 0.014a	0.75 ± 0.03a	0.25 ± 0.02a	70.6 ± 1.6a	24.3 ± 1.3a	38.4 ± 1.5a
**Zincol-2016**	Control	4.31 ± 0.1bc	0.36 ± 0.01 cd	0.49 ± 0.01bc	0.14 ± 0.01bc	53.3 ± 2.5bc	14.5 ± 0.6b	25.8 ± 0.5 cd
	STU	4.87 ± 0.2ab	0.38 ± 0.01bc	0.58 ± 0.04ab	0.18 ± 0.02ab	58.3 ± 2.3abc	17.6 ± 1.4b	30.1 ± 1.1bcd
	Co	3.06 ± 0.1d	0.24 ± 0.02e	0.40 ± 0.01c	0.07 ± 0.01c	34.3 ± 0.7d	8.33 ± 0.1c	14.9 ± 1.3e
	STU + Co	5.15 ± 0.2ab	0.41 ± 0.01abc	0.68 ± 0.02a	0.19 ± 0.02ab	63.6 ± 3.1ab	20 ± 1.3ab	33.0 ± 0.1abc

*STU* Sulfur rich thiourea, *CO* Cobalt stress, Difference among the letters after the values (means ± standard error of three replicates) shows a significant difference across the mean at *p* < 0.05 according to the Tukey HSD test

### Photosynthetic pigments

**Fig. 1:**
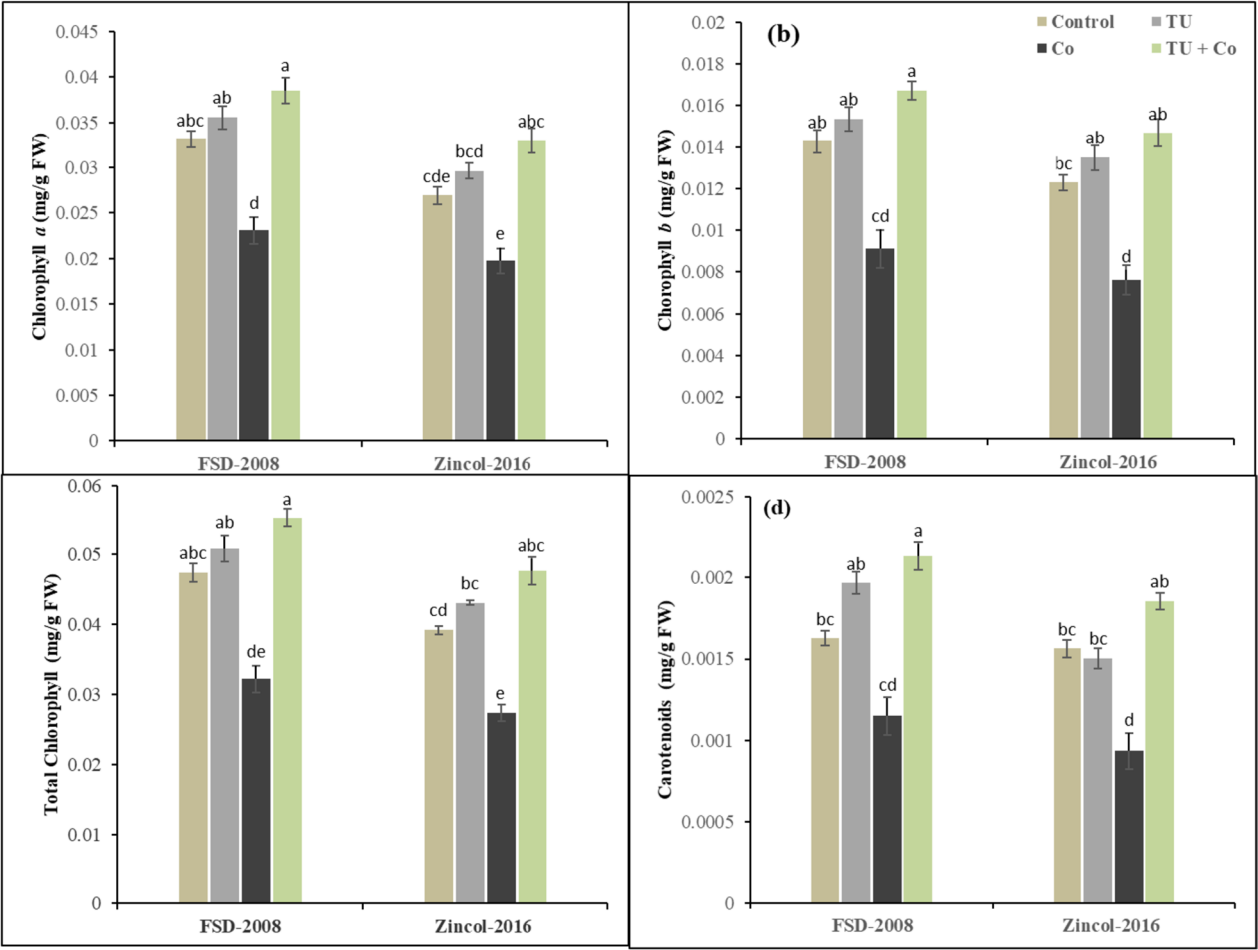
Influence of foliar applied STU on (**a**) Chlorophyll *a*, (**b**) Chlorophyll *b*, (**c**) Total chlorophyll, and (**d**) Carotenoids of both cultivars wheat under cobalt stress. The various letters above the mean show a significant difference across the mean at *p* < 0.05 according to the Tukey HSD test. Above the mean of three replicates the error bars show standard error (SE).

Photosynthetic pigments were significantly affected under the cobalt stress and STU foliar application in wheat varieties. The interaction among cobalt stress × STU applications × wheat varieties was non-significant for photosynthetic pigments. Results have revealed that cobalt stress decreased chlorophyll *a* (32%), chlorophyll *b* (47%), total chlorophyll (36%), and carotenoids by 46% in comparison to the control (Fig. 1). The wheat variety Zincol-2016 showed more reduction in photosynthetic pigments as compared to FSD-2008. However, STU foliar applications played an ameliorative role in reducing the detrimental effect of cobalt stress in wheat varieties and improved photosynthetic pigments. The total chlorophyll improved (16%) and (16%), and carotenoids (7%) and (15%) in wheat plants grown under cobalt stress. Among the cultivates, FSD-2008 showed better performance in comparison to Zincol-2016 under cobalt stress and STU applications in terms of photosynthetic pigments as total chlorophyll and carotenoids increased by (15%) and 15%) respectively in FSD-2008 as compared to Zincol-2016.

### Response of MDA and H_2_O_2,_ enzymatic antioxidants

**Fig. 2:**
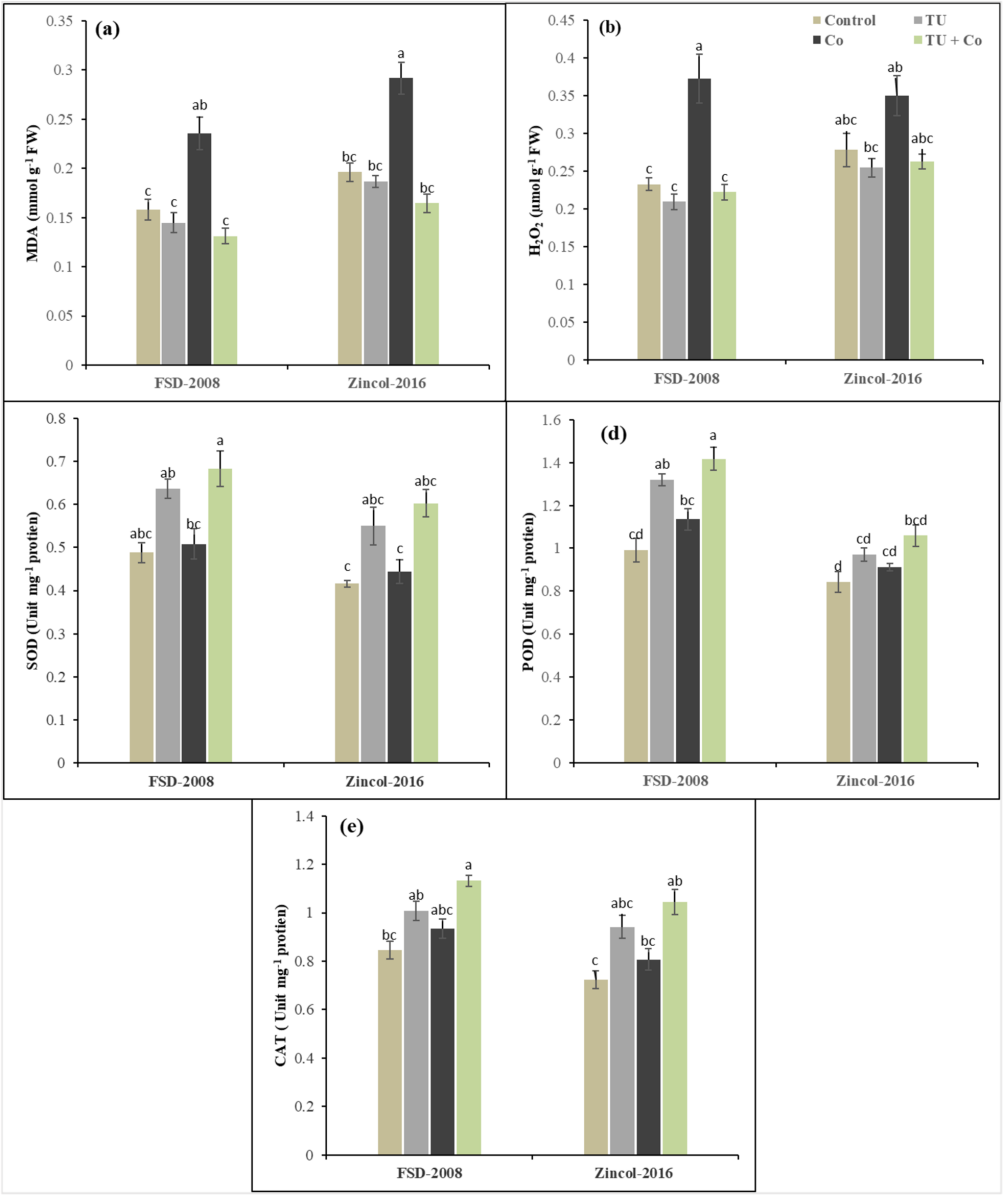
Influence of foliar applied STU on (**a**) MDA, (**b**) H_2_O_2,_ (**c**) SOD, (**d**) POD, and (**e**) CAT of both cultivars wheat under cobalt stress. The various letters above the mean show a significant difference across the mean at *p* < 0.05 according to the Tukey HSD test. Above the mean of three replicates the error bars show standard error (SE)

The activity of enzymatic antioxidants and reactive oxygen species was significantly affected under the cobalt stress and STU foliar application in wheat varieties. The interaction among cobalt stress × STU applications × wheat varieties was non-significant for enzymatic antioxidants and reactive oxygen species. Results have revealed that cobalt stress increased the SOD (5%), POD (9%), CAT (10%), MDA (10%) and H_2_O_2_ (11%) in comparison to control (Fig. 2). The wheat variety Zincol-2016 showed more response of H_2_O_2_ and MDA as compared to FSD-2008 while the activity of enzymatic antioxidants enhanced in FDS-2008 as compared to Zincol-2016. However, STU foliar application played an ameliorative role in reducing the detrimental effect of cobalt stress. The SOD significantly improved (30%) and POD (25%) in wheat plants grown under cobalt stress while through STU application the harmful effect of MDA was reduced (20%) and H_2_O_2_ (5%) which shows their effective role under stress conditions. Among the cultivates, FSD-2008 showed better performance in comparison to Zincol-2016 under cobalt stress and STU applications in terms of enzymatic oxidants as SOD and CAT increased (23%) and (10%) respectively in FSD-2008 as compared to Zincol-2016.

### Non-enzymatic antioxidants

**Fig. 3:**
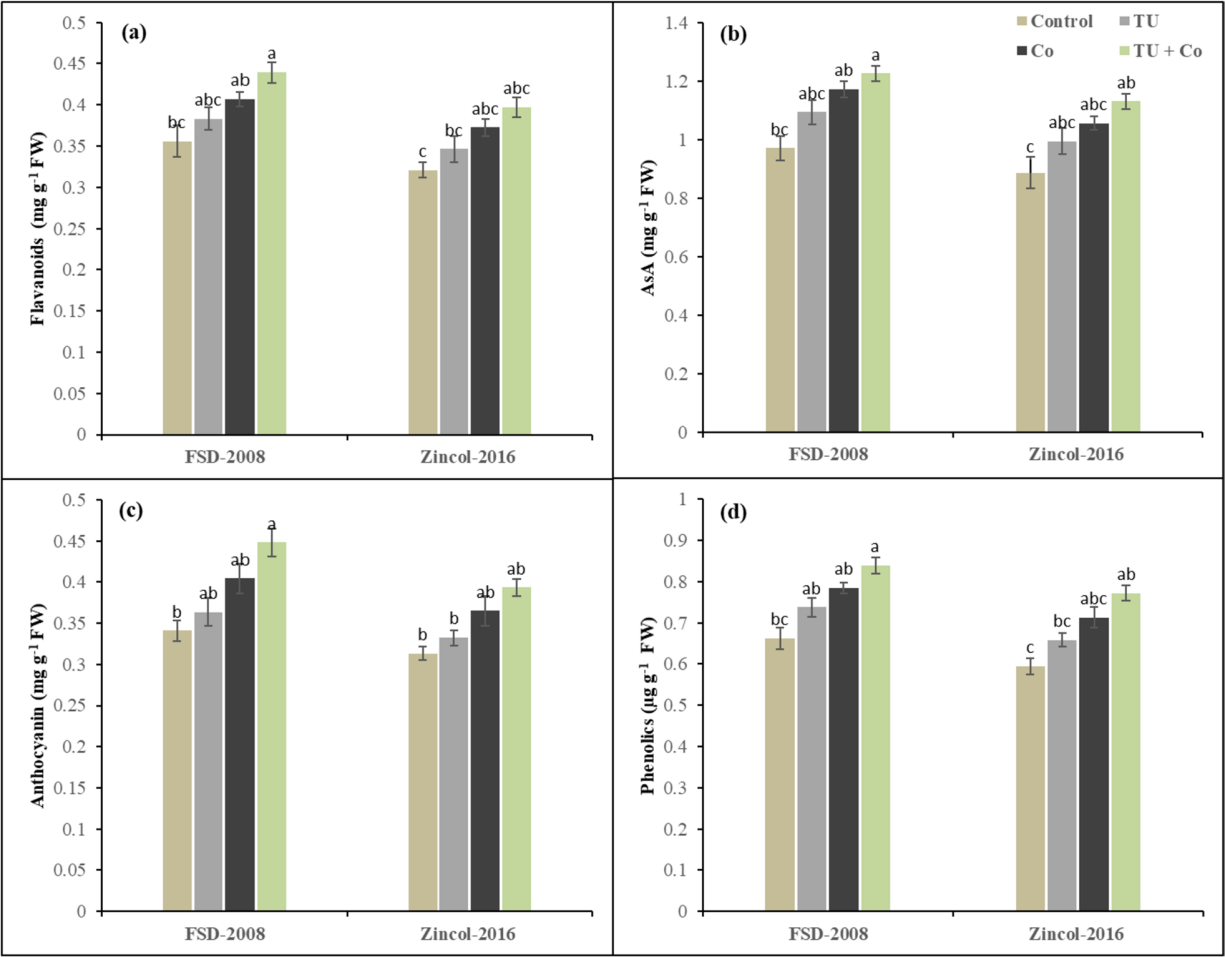
Influence of foliar applied STU on (**a**) Flavonoids, (**b**) Anthocyanin, (**c**) AsA, and (**d**) Phenolics of both cultivars of wheat under cobalt stress. The various letters above the mean show a significant difference across the mean at *p* < 0.05 according to the Tukey HSD test. Above the mean of three replicates the error bars show standard error (SE)

The activity of non-enzymatic was significantly affected under the cobalt stress and STU foliar application in wheat varieties. The interaction among cobalt stress × STU applications × wheat varieties was non-significant for non-enzymatic. Results have revealed that cobalt stress increased the flavonoids (13%), phenolics (16%), ascorbic acid (17%), and anthocyanin (16%) in comparison to control (Fig. 3). The wheat variety Zincol-2016 showed a decrease in non-enzymatic antioxidants as compared to FSD-2008. However, STU foliar application played an ameliorative role in reducing the detrimental effect of cobalt stress. The ascorbic acid significantly improved by 21% and anthocyanin by 22% in wheat plants grown in cobalt stress conditions. Among the cultivates, FSD-2008 showed better performance compared to Zincol-2016 under cobalt stress and STU applications in terms of non-enzymatic antioxidants as ascorbic acid and anthocyanin increased by 9% and 10% respectively in FSD-2008 as compared to Zincol-2016.

### Osmo-protectants

**Fig. 4:**
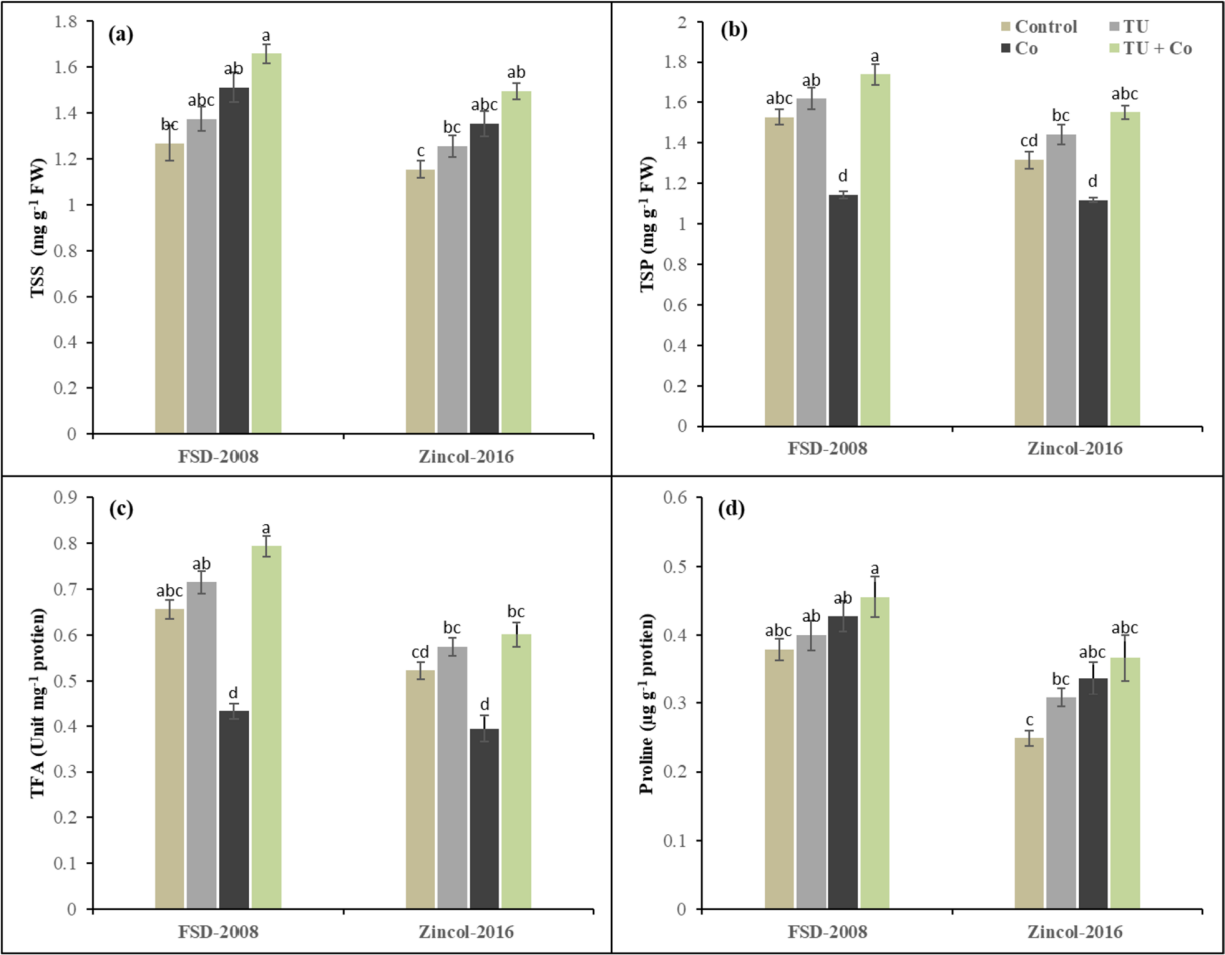
Influence of foliar applied STU on (**a**) TSS, (**b**) TSP, (**c**) TFA, and (**d**) Proline of both cultivars of wheat under cobalt stress. The various letters above the mean show a significant difference across the mean at *p* < 0.05 according to the Tukey HSD test. Above the mean of three replicates the error bars show standard error (SE)

Both wheat varieties show significant variation in the osmo-protectants level under the influence of cobalt stress and STU foliar application. The interaction among cobalt stress × STU applications × wheat varieties was non-significant for osmo-protectants. The results have revealed that cobalt stress increased TSS by (16%) and Proline by (19%) while decreasing the TSP (21%), and TFA (32%) in comparison to the control (Fig. 4). The wheat variety Zincol-2016 showed a decrease as compared to FSD-2008. However, STU foliar application plays a positive role in reducing the detrimental effect of cobalt stress. The proline improved (24%) and TSS (23%) in wheat plants grown under cobalt stress. Among the cultivates, FSD-2008 showed better performance as a comparison to Zincol-2016 under cobalt stress and STU applications in terms of osmo-protectants as proline and TSS increased (24%) and (9%) respectively in FSD-2008 as compared to Zincol-2016.

### Mineral nutrients

The ionic content for both varieties of wheat under the influence of cobalt stress and STU foliar application showed a significant variation. The interaction among cobalt stress × STU applications × wheat varieties was non-significant for ionic contents. The results have revealed that cobalt stress increased the sodium ions in root (13%), and shoot (16%) while reducing the potassium ions in roots (23%) and shoot (38%), calcium ions in roots (7%), and shoot (6%) and phosphorous content in roots (12%) and shoot (9%) as compared to control (Table 2). The wheat variety Zincol-2016 showed increased sodium ions while decreasing the potassium, calcium, and phosphorous contents as compared to FSD-2008. However, STU foliar application played an ameliorative role in reducing the detrimental effect of cobalt stress. The potassium ions increased in the shoot by 18% and phosphorous contents in the shoot (25%) in wheat plants grown under cobalt stress. Among the cultivates, FSD-2008 showed better performance in comparison to Zincol-2016 under cobalt stress and STU applications in terms of ionic contents as potassium ions in the shoot and phosphorous contents in the shoot increased (12%) and (34%) respectively in FSD-2008 as compared to Zincol-2016.

**Table 2:** Effect of foliar applied STU on the sodium, potassium, calcium and phosphorous level in the shoot and root of the wheat cultivars under cobalt stress conditions

Cultivars	Treatments	Shoot Na^+^ (mg/g FW)	Root Na^+^ (mg/g FW)	Shoot K^+^ (mg/g FW)	Root K^+^ (mg/g FW)	Shoot Ca^+2^ (mg/g FW)	Root Ca^+2^ (mg/g FW)	Shoot P (mg/g FW)	Root P (mg/g FW)
**FSD-2008**	Control	24 ± 1.61de	38.3 ± 1.12bcd	27 ± 0.89ab	49.6 ± 1.36bc	19.3 ± 0.68bc	26 ± 1.18bc	0.15 ± 0.007abc	0.08 ± 0.003ab
	STU	21 ± 0.89e	32.6 ± 1.36d	29 ± 0.93ab	57.6 ± 1.36ab	23 ± 0.89ab	30.6 ± 0.93ab	0.18 ± 0.005ab	0.09 ± 0.003ab
	Co	29 ± 0.89abc	42.6 ± 1.36bc	19 ± 1.1 cd	28.3 ± 1.36d	21 ± 0.89abc	28.3 ± 0.93abc	0.16 ± 0.007abc	0.06 ± 0.003 cd
	STU + Co	25 ± 1.61de	36.3 ± 1.86 cd	32.3 ± 1.18a	64.3 ± 1.4a	25.3 ± 0.68a	34.3 ± 0.68a	0.20 ± 0.005a	0.10 ± 0.002a
**Zincol-2016**	Control	33 ± 0.68ab	44.3 ± 1.12abc	23 ± 0.89bc	48.3 ± 1.36c	17 ± 0.89c	21.3 ± 0.93c	0.13 ± 0.006c	0.07 ± 0.003bc
	STU	28 ± 1.36bcd	40.6 ± 1.34bcd	26.3 ± 1.36ab	52.6 ± 1.43bc	19.3 ± 0.68bc	25 ± 0.89bc	0.15 ± 0.013bc	0.08 ± 0.002b
	Co	35 ± 0.68a	52.3 ± 1.36a	16 ± 1.6d	44.3 ± 1.4c	18 ± 0.44bc	23 ± 0.89c	0.14 ± 0.006bc	0.05 ± 0.004d
	STU + Co	30 ± 1.8abc	46.3 ± 1.69ab	29 ± 0.89ab	58.3 ± 1.36ab	22 ± 1.36abc	27 ± 0.89bc	0.18 ± 0.005ab	0.09 ± 0.002ab

*STU* Sulfur rich thiourea, *CO* Cobalt stress, Difference among the letters after the values (means ± standard error of three replicates) shows a significant difference across the mean at *p* < 0.05 according to the Tukey HSD test

### Correlation analysis and heat map

**Fig. 5:**
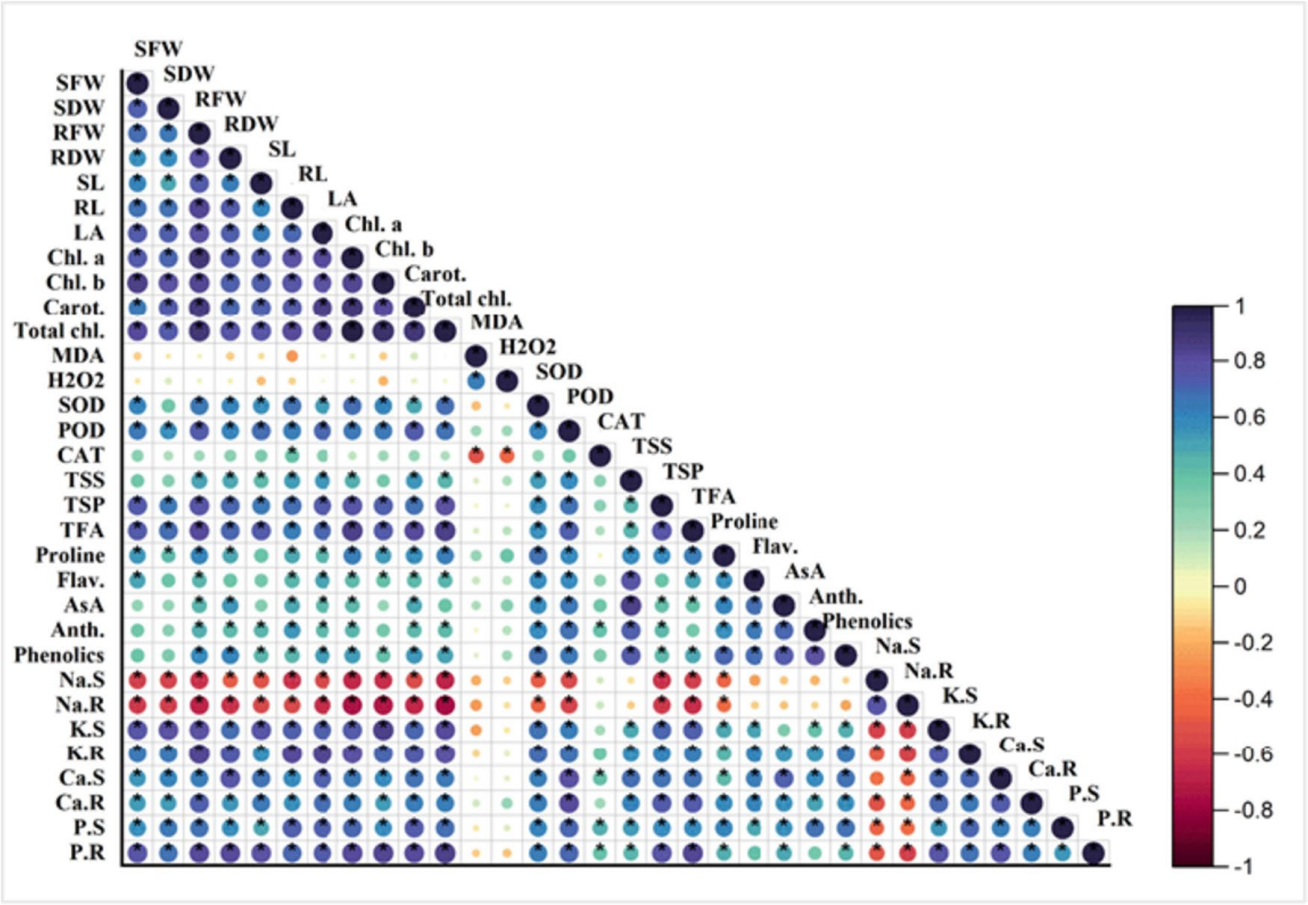
Correlation analysis between morpho-physiological, biochemical and ions attributes of both wheat cultivars. Abbreviations of the indices are SFW (Shoot fresh weight), SDW (Shoot dry weight), RFW (Root fresh weight), RDW (Root dry weight), SL (Shoot length), RL (Root length), LA (Leaf area), Chl. a (Chlorophyll *a*), Chl. b (Chlorophyll *b*), Total Chl. (Total chlorophyll), Carot. (Carotenoids), MDA (Malondialdehyde), H_2_O_2 (_Hydrogen peroxide), SOD (Superoxide dismutase), POD (Peroxidase), CAT (Catalase), TSS (Total soluble sugar), TSP (Total soluble protein), TFA (Total free amino acid), Proline, Flav. (Flavonoids), AsA. (Ascorbic acid), Anth. (Anthocyanin), Phenolics, Na.S (Shoot sodium), Na.R (Root sodium), K.S (Shoot potassium), K.R (Root potassium), Ca.S (Shoot calcium), Ca.R (Root calcium), P.S (Shoot phosphorous), and P.R (Root phosphorous)

Analysis of Pearson’s correlation shows strong negative and positive correlation in the indices of wheat cultivars measured under normal and stress conditions as shown in (Fig. 5). A strong positive correlation was observed in the morphological indices and photosynthetic pigments with potassium, calcium, and phosphorous ions in shots and roots while these indices showed a negative correlation with H_2_O_2_ and MDA. The TSP and TFA were positively correlated while the sodium ions in the shoots and roots of wheat cultivars showed a strong negative correlation. However, a slight positive correlation was observed in the enzymatic, non-enzymatic, and osmolytes in both cultivars of wheat.

**Fig. 6:**
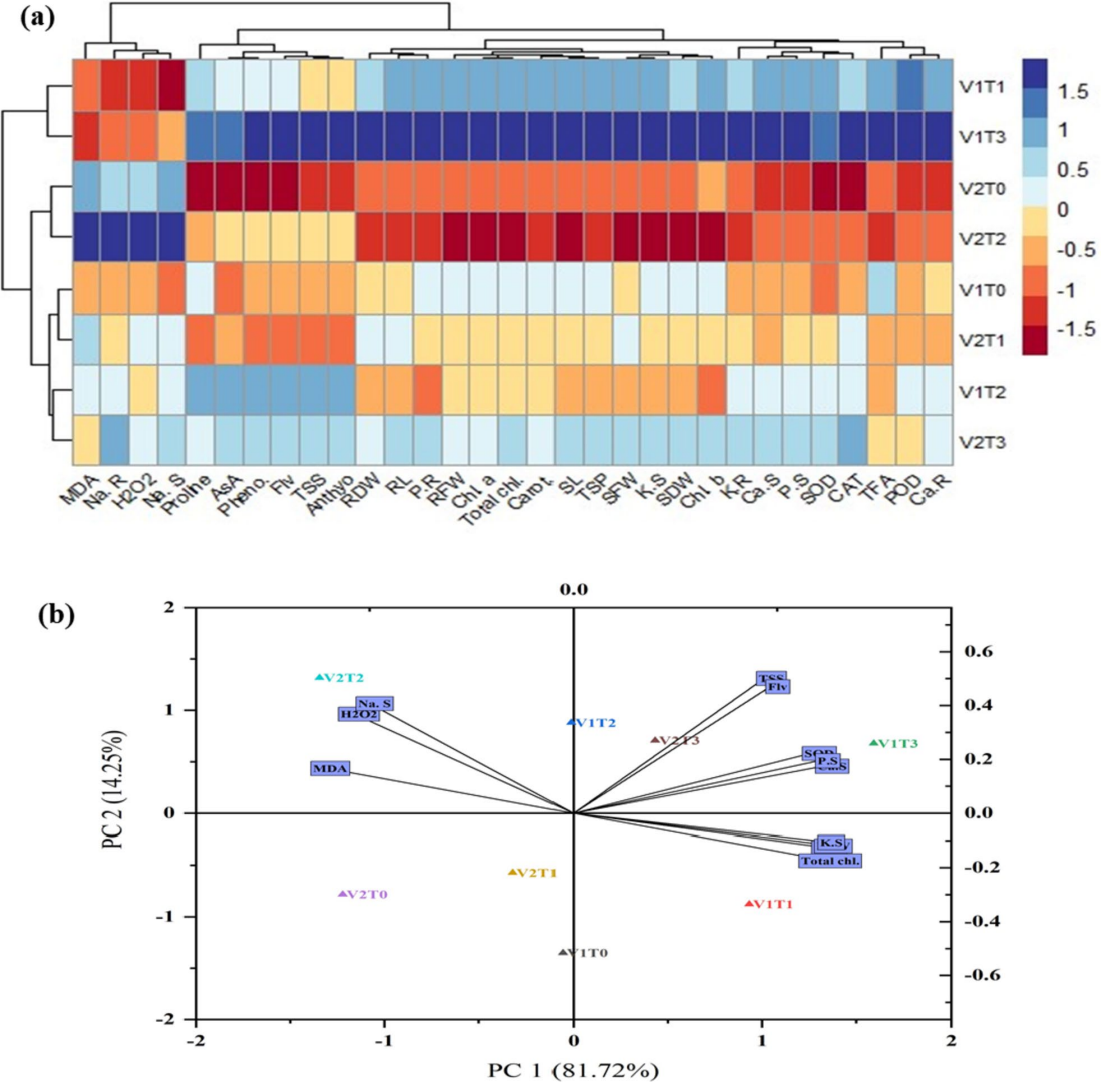
**a** Heat map and **b** Principal component analysis (PCA) showed the morphological, photosynthetic, biochemical, and ion contents in both cultivars of wheat. All measured indices abbreviations were seen in the caption of Fig. 5. while in the treatments group the T1, T2, T3, and T4 represent the control, TU-spray, cobalt stress and TU + cobalt stress respectively. The V1 and V2 represent the wheat cultivars, FSD-2008 and Zincol-2016 respectively

Analysis of the heat map was created across the morphological, photosynthetic, biochemical, and ion contents in both cultivars of wheat. The variation of colors in the boxes shows the interaction strength between the recorded above-mentioned indices and treatments. Scale colors from blue (strongly positive) to dark red (strongly negative) were closely correlated to the strength of the color gradient utilized in the heat map boxes. The highest enhancement was observed in the morphological indices, photosynthetic pigments, enzymatic and non-enzymatic antioxidants, osmolytes, phosphorous, potassium, and calcium ions while the lowest level of sodium ions and H_2_O_2_ and MDA in the treatment V1T3. In contrast, accumulation in the concentration of sodium ions and H_2_O_2_ and MDA was observed in the treatment V2T2 (Fig. 6).

## Discussion

Plant stress susceptibility is a crucial factor for plant maturation and development which stimulates signaling pathways and develops resistance to cope with stress [54]. In this experiment, we investigated the impacts of exogenous applications of STU at 500 µM on wheat cultivars grown under cobalt-induced toxicity. One of the major heavy metal stresses is cobalt stress [13, 15] which affects plant growth and yield by hurting plant gas exchange attributes, water status, plant biochemical parameters, and photosynthetic pigments. In this experiment, cobalt stress-induced toxicity caused a significant reduction in the performance of wheat cultivars by damaging photosynthetic pigments, reducing photosynthetic rate and plant water uptake, and affecting the plant physicochemical attributes (Tables 1 and 2; Figs. 2, 3, 4 and 5). The applications of STU played an ameliorative role under cobalt stress toxicity by upregulating plant defense systems against the damages caused by the accumulation of ROS and MDA.

The outcome of this study revealed that under cobalt stress, STU applications play a positive role in improving the morphological characteristics of wheat cultivars (Table 1). Cobalt-induced toxicity reduced the morphological parameters such as fresh and dry weight, and root and shoot length of both wheat cultivars as reported in the previous studies [55, 56] by reducing the nutrient uptake, plant water status, and stomatal conductance [14]. At the same time, the maximum reduction was observed in the wheat cultivar Zincol-2016 as compared to FSD-2008. However, exogenous applications of STU improved the plant growth indices including shoot and root length, and fresh and dry weight in both wheat cultivars grown under cobalt stress as found in the previous studies [57] while FSD-2008 showed maximum response towards STU and showed maximum improvement in growth parameters by detoxifying the cobalt stress-toxicity as compared to Zincol-2016. STU application may improve the leaf surface area which helps to capture more light, hence enhancing the carbon fixation that regulates assimilates production and splits them towards developing sinks enhancing crop development and yield parameters. Under stress conditions, thiourea enhanced the osmotic capacity by improving the cell turgidity that allowed the plant to hold its water balance to raise the transpiration rate which in turn improved the morphological indies in the plants. Foliar applications of STU at 500 µM concentration improved plant growth and development under stressed conditions by improving the length of root and shoot and dry matter production [24, 34, 58].

In the current study, we noted remarkable changes across the wheat cultivars for their photosynthetic pigment accumulation responses to cobalt stress-induced damages to the photosynthetic apparatus. Both wheat cultivars grown under cobalt stress showed an overall decrease in photosynthetic pigments (Fig. 2) for instance carotenoid content, total chlorophyll, and chlorophyll *a*, and *b* as reported in the studies [59, 60]. The maximum reduction in photosynthetic pigments was noted in the wheat cultivar Zincol-2016 and the minimum reduction was noted in FSD-2008 due to its better genetic potential and resilient ability under stressful conditions. Oxygen radicle generation damages the cell membrane under cobalt stress, leading to the breakdown of chloroplasts or the manufacture of intermediary products in the process leading to the creation of carotenoids and chlorophyll. Because of high Co redox potential and inhibition of the enzymatic mechanisms responsible for chlorophyll production, there may be a correlation between the drop in chlorophyll concentration under stressful circumstances and the reductive steps inhibited in the chlorophyll biosynthetic pathway. Under cobalt stress, the creation of oxygen radicals damages the membrane of the cell, leading to the breakdown of chloroplasts or the biosynthesis of intermediate products in the process leading to the development of carotenoids and chlorophyll [60–62]. However, STU foliar applications improved the formation of photosynthetic pigments which play a positive role in the photosynthetic efficiency that is essential for the development and growth of both wheat varieties while FSD-2008 showed maximum improvement in the photosynthetic pigments as compared to the Zincol-2016 as reported in the previous investigations [63–65]. The oxidative damage of photosynthetic pigments especially carotenoids and chlorophyll levels lowered by the application of STU in both varieties of wheat under Co-stress [66]. Thiourea usage may result in an upsurge in photosynthesis due to its role in ferredoxin that promotes synthesis and sustaining chlorophyll contents thereby boosting the photosynthetic rate and absorption efficiency that subsidizes the development and growth of plants [67].

To induce toxicity at the cellular level cobalt stress inhibits plants from developing and growing by increasing the ROS (free radicles) amount is the most popular way of revealing oxidative damage at the cellular level [68, 69]. Results have revealed increased ion accumulation of MDA and H_2_O_2_ levels in wheat varieties under cobalt stress conditions, this led to a significant reduction in cell membrane stability which consequently raised the metabolite leakage in intercellular spaces from cells [70]. The maximum accumulation of MDA and H_2_O_2_ was observed in the wheat cultivar Zincol-2016 as compared to FSD-2008 as observed in the studies [71, 72]. During oxidative stress, the generation of reactive oxygen species causes severe damage to organelles and membrane stability [73]. However, STU-based anti-oxidative machinery shielded the plant from this oxidative stress. Thiourea application significantly increased the amount and efficiency of TU-mediated non-enzymatic antioxidants (ASA, Phenolic, Anthocyanin, and Flavonoids), enzymatic antioxidants (SOD, POD, and CAT), and osmolyte/ metabolite production (Proline, TFA, TSS, and TSP) that protects the plant from serious damages by detoxification mechanism [74, 75]. Results have revealed that foliar applications of STU improved the defense system and reduced the production of H_2_O_2_ and MDA of wheat varieties grown in stressed environments by upregulating the activities of CAT, POD, SOD, ASA, Phenolic, Anthocyanin, Flavonoids, Proline, TFA, TSS, and TSP, while wheat cultivar FSD-2008 showed maximum response towards STU and showed maximum improvement in antioxidant defense system by detoxifying the cobalt stress-toxicity as compared to Zincol-2016 as observed in the previous studies [23, 76, 77]. While across the cultivars either sensitive or tolerant have metabolic pathways in synthesizing detoxifying chemicals for ROS. The difference lies in how plants accumulate these compounds and avoid cellular damage to function under demanding conditions (Figs. 3, 4 and 5). STU enhances cell membrane stability by suppressing MDA and a direct ROS scavenger that neutralizes endogenous H_2_O_2_ [78, 79]. The redox state of membrane proteins is maintained via thiourea by quenching the oxygen radicles generated under cobalt stress.

The thiourea-based antioxidative machinery (–SH group) comprises, GSH (glutathione), proteins, certain amino acids, and enzymatic and non-enzymatic antioxidants that serve as a stress response [21, 80], the plant physiological processes up-regulated by these antioxidants. The redox state in plant cells improved by thiourea application, modulating antioxidant activities, and resulting in the lowering of lipid peroxidation products [81]. Due to this application, the damages of abiotic stress on plants are mitigated by improving plant metabolism. This was attained by managing photosynthetic efficiency and photosynthetic pigments [60], which is crucial for redox control during oxidative stress homeostasis along with physiological and phonological development [81]. In plants, the immediate stress-induced response is proline accumulation which serves as a selectable trait useful for evaluating abiotic stress resistance [82]. The improvement in activities of SOD plays a crucial role in converting O_2_^∙−^ radicals to H_2_O_2_ and O_2_ as the first line of defense against oxidative damage to cells [83]. In addition, CAT takes part in the process of converting H_2_O_2_ into H_2_O as well as being essential for plant metabolism and signal perception [33]. Proline is generated under stress due to higher synthesis and slower degradation rate [84]. Proline acts as an osmolyte that functions as a signaling molecule under stressed conditions, scavenges reactive oxygen, sustains sub-cellular structures, and improves the redox state [85]. Thiourea accumulates osmolytes which play a vital role in plant growth, gaseous exchange upregulation, and plant water status. Thiourea controls the proteins and sugar accumulation along with nitrogen metabolism formation. It showed that under stress, thiourea plays a possible role in mitigating the adverse effect of cobalt stress in wheat plants as shown in (Figs. 5 and 6).

The outcome of the research revealed that cobalt stress and TU applications positively affected the nutrient absorption in root and shoot on a fresh weight basis in wheat cultivars (Table 2). Results have revealed that Na^2+^ accumulation in roots and shoots was significantly increased in both wheat varieties under cobalt stress, even though the K^+^, Ca^2+^, and P accumulation decreased as found in the investigation [56]. The toxic metals limit the minerals and water uptake in plants and are intimately linked with their widespread accumulation, particularly in roots, and also prevent the entry and binding of essential ions like K, Ca^2+,^ and Mg^+^ [86, 87]. However, STU applications played an ameliorative role and reduced the concentration of Na^2+^ in wheat roots and shoots, but in contrast, STU improved the accumulations of P, K^+^, and Ca^2+^ significantly as compared to the control. The highest improvement in nutrient accumulations was observed in FSD-2008 as compared to Zincol-2016 under STU applications and cobalt stress. Cobalt stress reduced the transpiration rate and stomatal conductance which do not permit the water uptake by the roots thus reducing the uptake of nutrients. However, thiourea played an important role in regulating nutrient uptake by improving plant water status and stomatal conductance [36].

## Conclusion

The potential of sulfur-rich thiourea (STU) treatment for mitigating the detrimental effects of cobalt stress on wheat cultivars was demonstrated, and its effectiveness at a low concentration of 500 µM. Results have revealed that cobalt stress can negatively impact the growth and development of wheat cultivars by improving the production of MDA and H_2_O_2_. In addition, Na concentration was also maximized in both wheat varieties under cobalt stress. However, STU application ameliorated the damages caused by cobalt stress in wheat cultivars by upregulating the plant defense system (antioxidant activities and osmolyte productions). In particular, thiourea treatment can improve plant growth by improving the photosynthetic pigments and nutrient uptake under cobalt stress. The study indicates that thiourea supplementation at the growth stage may enhance the performance of wheat plants, especially under cobalt metal stress by increasing secondary metabolites and osmolytes, STU enhances nutrient absorption, controls metabolic processes, and promotes tolerance in stressful conditions. However, more extensive fieldwork is required to corroborate this idea. Along with all these findings, there is a dire need to study the effect of STU applications on plant signaling mechanisms and their role at the cell level. For a better comprehension of the function of STU in plant signaling networks under stress, more investigations utilizing histology, proteomics, and genetic data are required. Verification of STU function as a synchronizer for many plant functions, such as development, hormone regulation, and the synthesis of secondary metabolites with the present scenario of global climate change, it is necessary to design a sustainable, economical, and workable solution for the STU application to reduce crop losses in stressful situations and improve agricultural plant production potential.

## Data Availability

All data generated or analyzed during this study are included in this published article.
